# Simulated Microgravity Remodels Extracellular Matrix of Osteocommitted Mesenchymal Stromal Cells

**DOI:** 10.3390/ijms22115428

**Published:** 2021-05-21

**Authors:** Ivan Zhivodernikov, Andrey Ratushnyy, Ludmila Buravkova

**Affiliations:** Institute of Biomedical Problems, Russian Academy of Sciences, Khoroshevskoye Shosse, 76a, 123007 Moscow, Russia; kordait-2213@yandex.ru

**Keywords:** mesenchymal stem cells (MSCs), osteogenesis, simulated microgravity (SMG), random positioning machine (RPM), extracellular matrix (ECM), proteases

## Abstract

The extracellular matrix (ECM) is the principal structure of bone tissue. Long-term spaceflights lead to osteopenia, which may be a result of the changes in composition as well as remodeling of the ECM by osteogenic cells. To elucidate the cellular effects of microgravity, human mesenchymal stromal cells (MSCs) and their osteocommitted progeny were exposed to simulated microgravity (SMG) for 10 days using random positioning machine (RPM). After RPM exposure, an imbalance of MSC collagen/non-collagen ratio at the expense of a decreased level of collagenous proteins was detected. At the same time, the secretion of proteases (cathepsin A, cathepsin D, MMP3) was increased. No significant effects of SMG on the expression of stromal markers and cell adhesion molecules on the MSC surface were noted. Upregulation of *COL11A1*, *CTNND1*, *TIMP3*, and *TNC* and downregulation of *HAS1*, *ITGA3*, *ITGB1*, *LAMA3*, *MMP1*, and *MMP11* were detected in RPM exposed MSCs. ECM-associated transcriptomic changes were more pronounced in osteocommitted progeny. Thus, 10 days of SMG provokes a decrease in the collagenous components of ECM, probably due to the decrease in collagen synthesis and activation of proteases. The presented data demonstrate that ECM-associated molecules of both native and osteocommitted MSCs may be involved in bone matrix reorganization during spaceflight.

## 1. Introduction

The extracellular matrix (ECM) comprises a significant part of connective tissues and determines their mechanical properties. Mechanical strength is of great importance for all connective tissues. This is one of the reasons to pay attention to peculiarities of the ECM under microgravity conditions. Connective tissue ECM is formed mainly by two types of macromolecules, fibrillar proteins and proteoglycans. The first are represented by several types of collagens and elastin. Collagen I is the main structural component of bone tissue that provides its tensile strength [1]. Proteoglycans consist of proteins and glycosaminoglycans (GAGs), non-branching polysaccharide molecules that occupy a large volume and are unable to fold into globules such as proteins. These molecules carry negative charges and, therefore, they can retain a large amount of osmotically active ions and water, which creates a high turgor pressure of the ECM and its compressive strength. Due to proteoglycans, ECM deposits numerous growth factors that belong to the FGF, VEGF, TGF, etc., families, thus ensuring their stable levels in the pericellular space or the release of large amounts during degradation [2,3]. Proteoglycan levels determine matrix mineralization and collagen fiber diameters in bone tissue [4].

ECM components are associated with the cytoskeleton through cell adhesive structures, whereby mechanical stress is transmitted in the intercellular space and inside the cells, affecting cellular signaling and gene expression [5]. The ECM continuously undergoes structural changes through synthesis, degradation, mineralization, and other processes [6]. These processes depend on various factors, the most important being the mechanical load or its critical decrease in spaceflight. For example, a decrease in bone mineral density, which is associated with the reorganization of ECM, was detected after long-term spaceflights [7,8,9].

Osteopenia in microgravity is caused by both systemic and cellular factors. Currently, a decreased support loading, redistribution of body fluid pressure, osteoclast activation, reduction in osteoblast proliferation and differentiation, and lysis of osteocytes are considered to be the main factors in the development of osteopenia [10]. Moreover, a loss of bone tissue calcium is aggravated by parathyroid hormone regulation, resulting in reduced calcium reabsorption in the kidneys [7,11]. To study the effects of microgravity on stromal cells, different cell types were applied as well as different approaches to simulate microgravity [12,13,14,15,16].

The current concept suggests that impaired cell physiology under microgravity is the trigger of negative changes in bone tissue [17,18]. This is supported by numerous data from in vitro experiments conducted aboard space vehicles and under ground-based microgravity simulations. The reduced osteogenic potential of osteoblasts and low commitment of stromal progenitors in vitro has been convincingly demonstrated [19,20,21,22]. In addition, a downregulation of osteogenic-associated genes and upstream signaling pathway genes has been observed [23,24]. The changes in ECM composition during osteogenic differentiation may alter the mechanical properties of the tissue and also affect certain signaling pathways involved in osteogenic commitment [25,26,27].

Mesenchymal stromal cells (MSCs) participate in the maintenance of homeostasis in many tissues, including bone. Being osteoblast precursors, MSCs actively participate in ECM production and remodeling, which have been investigated for medical purposes [28,29,30]. These cells are important for proper execution of osteogenesis and response to microgravity. Under simulated microgravity, MSCs have demonstrated changes in functional state, response to inflammatory stimulation, the production of many proteins, and gene expression [31,32]. Multidirectional changes in the transcription of genes encoding ECM proteins and associated molecules were found in response to SMG. The transcriptomic shifts depended on cell type and exposure time [33,34,35,36].

An upregulation of *COL4A5* and the main matrix glycoprotein, fibronectin (*FN*), as well as enzymes involved in ECM remodeling (*MMP1* and *MMP3*) was found after 3 days of RPM exposure of fibroblasts [37]. After 7 days of 2D clinorotation, an increased expression of *COL1* and *COL3* was detected in MSCs [36]. On the other hand, there are data on downregulation of *Col1a* and *Fbn1* (fibrillin) following 7-day clinorotation [36,38]. Regardless of the mode and exposure time, a decreased transcription of osteomodulin (*OMD*) that regulates osteoblast adhesion was demonstrated for preosteoblasts [20,39]. The available data do not allow drawing certain conclusions about the patterns of ECM remodeling. Meanwhile, the experimental data in this field could expand the knowledge of how the activity of stromal cells determines changes in bone tissue under microgravity. The purpose of this study was to analyze the peculiarities of ECM changes under the influence of simulated microgravity on MSCs in vitro.

## 2. Results

### 2.1. MSC Characterization

**Figure 1:**
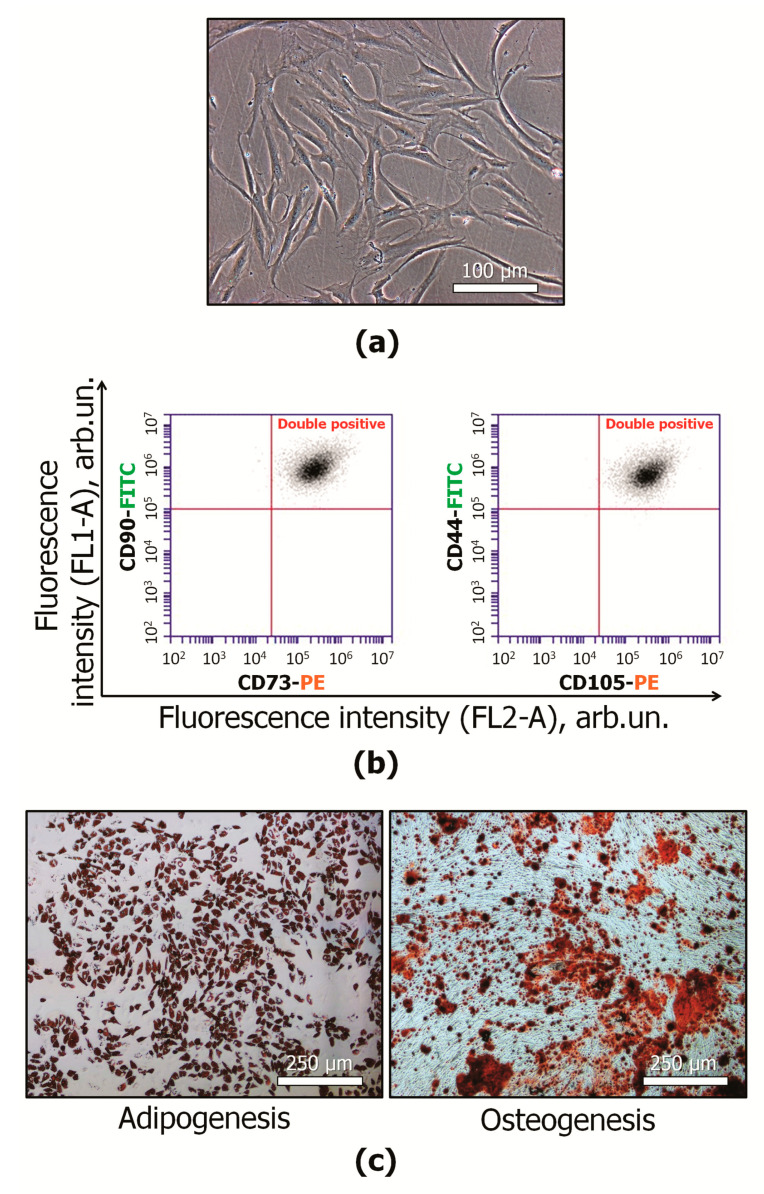
Characterization of MSCs: (**a**) representative image of MSCs in vitro, light microscopy (phase contrast); (**b**) representative flow cytometric plots of immunophenotype. MSCs were stained with antigen specific fluorescent antibodies: CD44-FITC, CD73-PE, CD90-FITC, CD105-PE; (**c**) confirmation of multilineage potential of MSCs, representative images, light microscopy (bright-field). MSCs cultured in adipogenic differentiation medium were stained with oil red O. MSCs cultured in osteogenic differentiation medium were stained with alizarin red S. MSCs—mesenchymal stromal cells; FITC—fluorescein isothiocyanate; PE—phycoerythrin.

Primary MSCs (3–4 passages) satisfied the minimal criteria of the International Society for Cell and Gene Therapy (ISCT) statements [40]. They had fibroblast-like morphology (Figure 1a). The cells were positively stained with fluorescent antibody against some stromal markers: CD29+, CD73+, CD90+, CD105+ (Figure 1b). Osteogenic and adipogenic differentiation in the presence of the appropriate stimuli was shown (Figure 1c).

### 2.2. MSC Commitment to the Osteoblast Lineage

**Figure 2:**
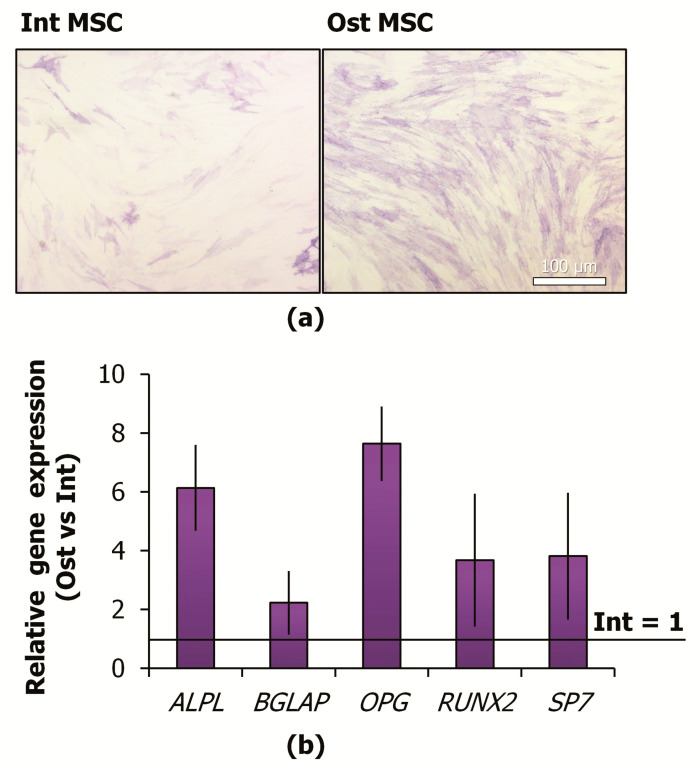
Confirmation of MSC osteogenic commitment (7-day induction): (**a**) representative images of histochemical detection of ALP activity and dye optical density of alkaline phosphatase activity staining, light microscopy (bright-field); (**b**) relative expression of osteogenesis-associated genes (Ost vs. Int), qPCR analysis. MSCs—mesenchymal stromal cells; Int—intact MSCs; Ost—osteocommitted MSCs.

Inducers of osteogenic differentiation were added to the culture medium for 7 days. Increased activity of alkaline phosphatase was shown using a cytochemistry method (Figure 2a). Alkaline phosphatase catalyzes the hydrolysis of organic phosphate esters in the extracellular space. An increase in the activity of this enzyme is one of the early signs of MSC osteo-differentiation. At the same time, after 7-day exposure, MSCs retained the progenitor properties and did not show signs of matrix mineralization that is the typical marker of the late stage of osteogenic differentiation [41].

Quantitative PCR (qPCR) analysis revealed more than 2-fold upregulation of osteogenesis-associated genes (*RUNX2*, *ALPL*, *OPG*, *SP7*, *BGLAP*) (Figure 2b). RUNX2 (runt-related transcription factor 2) is a key positive regulator of osteoblast differentiation. *ALPL* (alkaline phosphatase), *OPG* (osteoprotegerin), *SP7* (osterix), *BGLAP* (osteocalcin) are the protein-coding genes associated with osteogenesis.

### 2.3. SMG Effects on Immunophenotype and Adhesion Molecules

**Figure 3:**
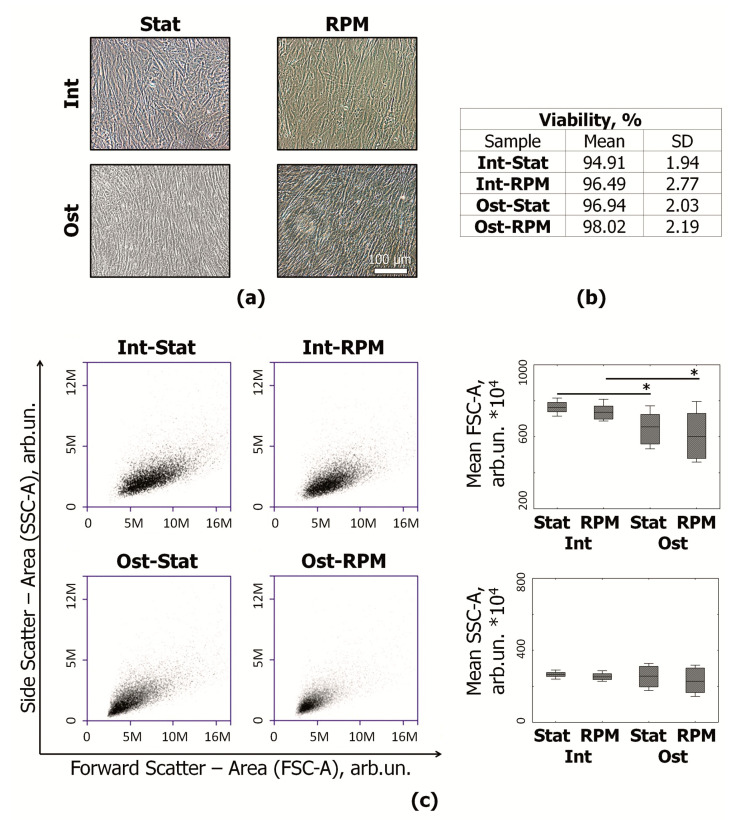
Morphology and viability of MSCs after 10 days of RPM exposure: (**a**) representative images of MSCs, light microscopy (phase contrast); (**b**) the share of viable MSCs; (**c**) evaluation of MSC size/granularity. Representative flow cytometry plots of FSC-A/SSC-A (size/granularity, respectively) distribution and boxplots. Data are presented as median, Q1/Q3, min/max; *n* ≥ 10, * *p* < 0.05. MSCs—mesenchymal stromal cells; Int—intact MSCs; Ost—osteocommitted MSCs; Stat—static control; RPM—random positioning machine; FSC-A—forward scatter area; SSC-A—side scatter area.

The flasks with osteocommitted (Ost) and intact (Int) MSCs were placed on the random positioning machine (RPM) for 10 days. A part of the flasks was left as a static control (Stat). During the experiment, the cells retained their adhesive properties. In all experimental groups, the MSCs formed a dense cell monolayer (Figure 3a). Cell viability, morphology, and immunophenotype were studied using flow cytometry. Viability assessment did not reveal the significant cell death under simulated microgravity (Figure 3b). The share of living MSCs remained high in all samples (≥94%). To evaluate the cell size and granularity, forward scatter (FSC) and side scatter (SSC), respectively, were analyzed (Figure 3c). It was shown the average size of osteocommitted cells is significantly smaller than that of intact cells. Cytoplasmic granularity did not differ significantly between the experimental groups.

**Figure 4:**
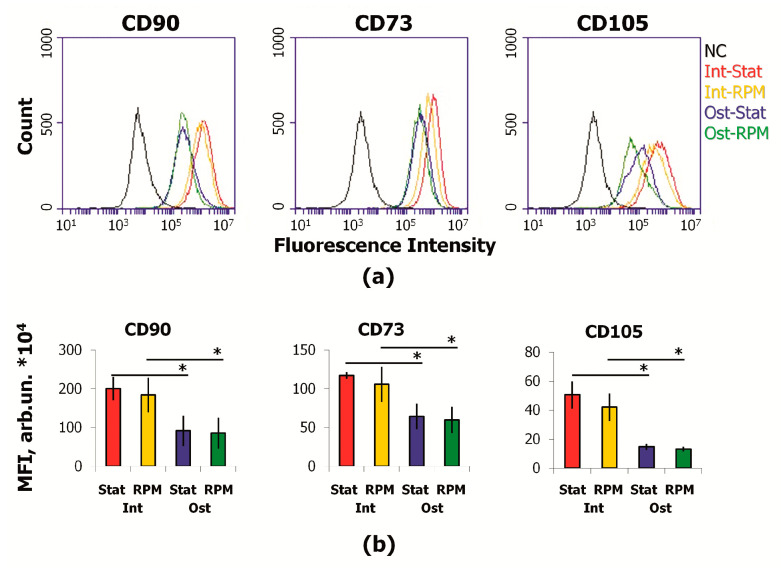
Immunophenotype of MSCs after 10 days of RPM exposure: (**a**) representative fluorescence plots; (**b**) mean fluorescence intensity (MFI) of CD90, CD73, and CD105. Data are presented as mean ± SD; *n* ≥ 3, * *p* < 0.05. MSCs—mesenchymal stromal cells; Int—intact cells; Ost—osteocommitted cells; Stat—static control; RPM—random positioning machine; NC—negative control (MSCs were stained with matched fluorescent nonimmune antibody).

**Figure 5:**
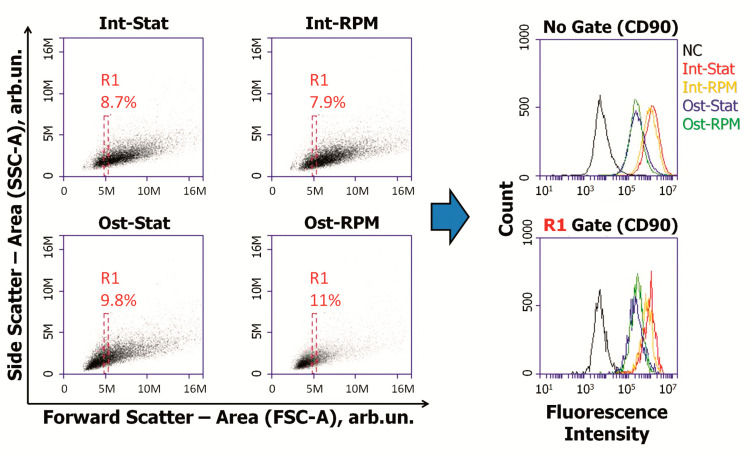
Mean fluorescence intensity of specific antibody to CD90, representative experiment. R1 gate included MSCs of the same size, but differentiated fluorescence remained. MSCs—mesenchymal stromal cells; Int—intact cells; Ost—osteocommitted cells; Stat—static control; RPM—random positioning machine; NC—negative control (MSCs were stained with matched fluorescent nonimmune antibody); FSC-A—forward scatter area; SSC-A—side scatter area.

Analysis of the main MSC markers showed a decrease in CD73, CD90, CD105 expression during osteocommitment (Figure 4). This effect was not associated with the average cell size reduction. To demonstrate this, we gated cells of the same sizes (R1) and compared gated MSCs in experimental groups (Figure 5). Despite the same sizes in the R1 gates, a significant difference in the expression of stromal markers persisted.

**Figure 6:**
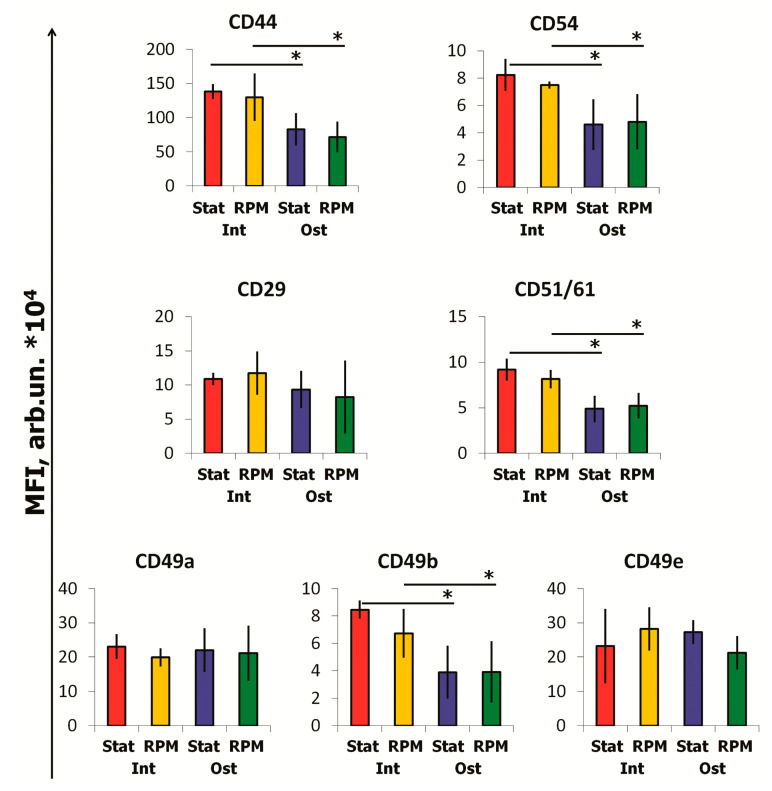
Expression of adhesion molecules on MSCs after 10-days of RPM exposure, flow cytometry. Data are presented as mean ± SD; *n* ≥ 3, * *p* < 0.05. MSCs—mesenchymal stromal cells; Int—intact cells; Ost—osteocommitted cells; Stat—static control; RPM—random positioning machine; MFI—mean fluorescence intensity.

Osteocommitment led to decreased expression of CD44 (HCAM), CD54 (ICAM), CD51/61 (integrin αv/β3), CD49b (Integrin α2) on cell membranes (Figure 6). At the same time, the expression of CD29 (Integrin β1), CD49a (Integrin α1), and CD49e (Integrin α5) did not change.

No significant effects of 10-day SMG on the expression of stromal markers and cell adhesion molecules were found. Interestingly, that short-term (96 h) exposure of MSCs under SMG reduced the expression of CD105, CD51/61, CD44, and CD54 and altered the transcription activity of several genes associated with adhesion and extracellular matrix [42]. Probably, longer exposure allows MSCs to adapt to SMG and restore the normal expression of certain surface molecules.

### 2.4. SMG Effects on ECM Proteins

**Figure 7:**
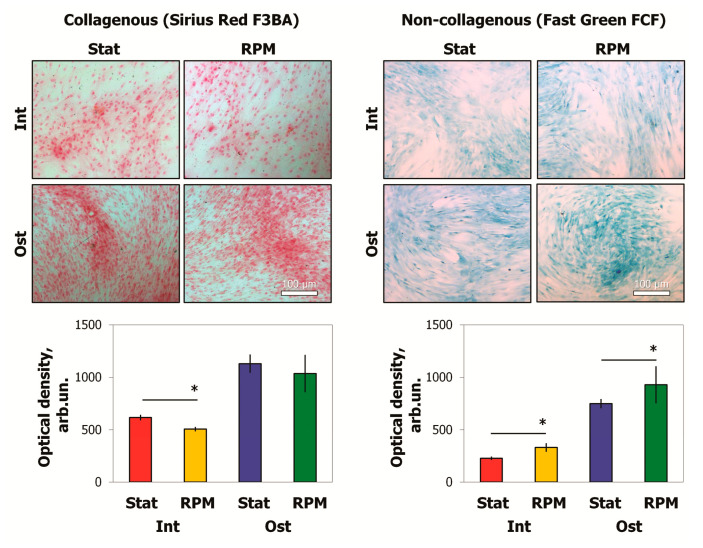
Evaluation of collagenous and non-collagenous proteins of MSCs after 10 days of RPM exposure, light microscopy (bright field), and spectrophotometry. Sirius Red F3BA and Fast Green FCF selectively bind collagenous and non-collagenous components of ECM, respectively. Data are presented as mean ± SD; *n* ≥ 3, * *p* < 0.05. MSCs—mesenchymal stromal cells; Int—intact MSCs; Ost—osteocommitted MSCs; Stat—static control; RPM—random positioning machine; ECM—extracellular matrix.

To assess the content of ECM components after 10 days of RPM exposure, we used Sirius Red F3BA and Fast Green FCF dyes, which selectively bind to collagenous and non-collagenous proteins, respectively. Then, the dyes were extracted, and the optical densities of the solutions were analyzed (Figure 7). The results showed an increase in ECM non-collagenous proteins after RPM exposure. These proteins were increased up to 35% in the intact MSCs, while the osteo-MSCs demonstrated a 20% increase. A 15% decrease of ECM collagen components was shown in intact cells after RPM exposure. Some tendency was found in osteocommitted MSCs, but the changes were not significant.

**Figure 8:**
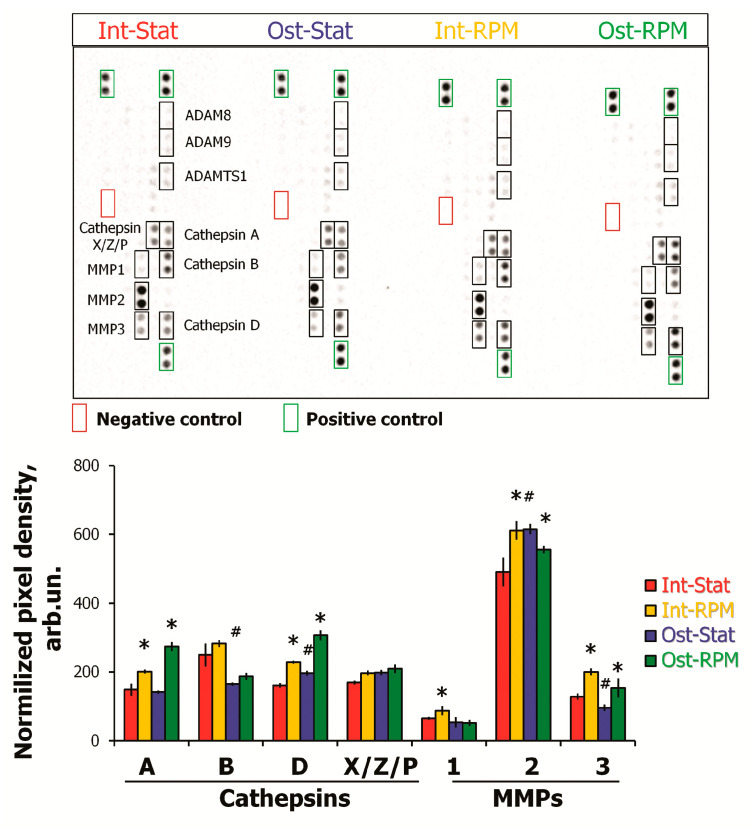
Evaluation of protease levels in conditioned medium of MSCs after 10 days of RPM exposure using Proteome Profiler Human Protease Array Kit. Representative image of the dot blotting of protein levels is shown. Densitometric analysis of certain analytes was performed using ImageLab software (Bio-Rad, Hercules, CA, USA). The major proteins are demonstrated. The data were normalized to positive control and are shown as mean ± SD; *n* ≥ 4, * *p* < 0.05 (RPM vs. Stat; # Ost-Stat vs. Int-Stat). MSCs—mesenchymal stromal cells; Int—intact cells; Ost—osteocommitted cells; Stat—static control; RPM—random positioning machine.

The conditioned medium was analyzed using Proteome Profiler Human Protease Array Kit. The 7 days of osteocommitment lead to decreased production of cathepsin B and MMP3 (matrix metalloproteinase-3) and increased cathepsin D (Figure 8). Both intact and osteo-MSCs demonstrated the alteration of protease production in a similar manner under SMG. Increased secretion of cathepsin A, cathepsin D, and MMP3 was detected. Interestingly, RPM exposure resulted in an enhanced level of MMP2 in intact MSCs, while the reverse effect was observed in osteocommitted cells (Figure 8).

It should be noted that the MMP3 level decreased in osteocommitted MSCs, but simulated microgravity led to enhanced secretion of this protease (Figure 8). The MMP3 can degrade laminin, fibronectin, several gelatins, collagens III, IV, X, and IX, and cartilage proteoglycans.

### 2.5. SMG Effects on Gene Expression

**Figure 9:**
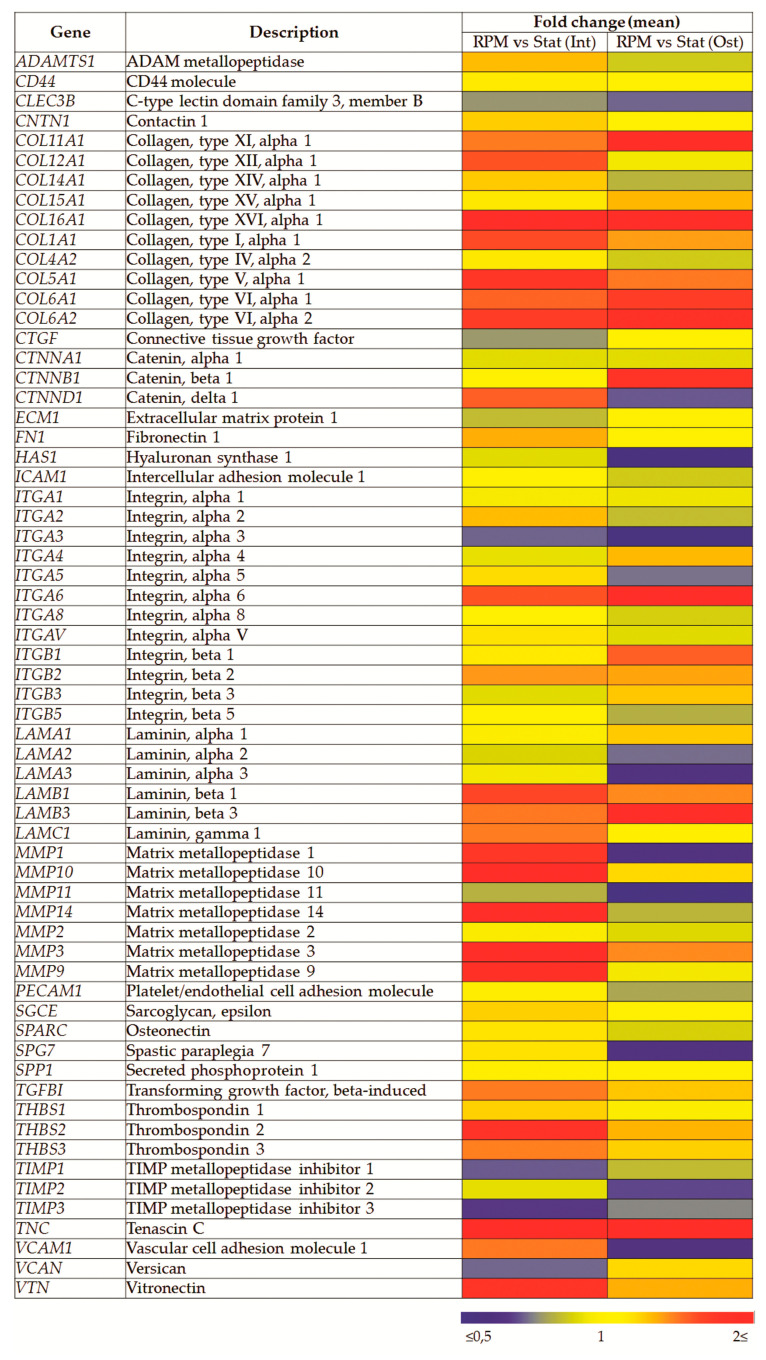
Relative expression of matrix/adhesion associated genes after 10 days of RPM exposure. Heatmap, *n* ≥ 3. Int—intact cells; Ost—osteocommitted cells; Stat—static control; RPM—random positioning machine.

After 10 days of RPM exposure, the expression of 84 genes associated with adhesion and matrix production was analyzed. This gene profiler included cell adhesion molecules (transmembrane receptors, cell–cell adhesion, cell–ECM adhesion, other cell adhesion molecules) and ECM molecules (basement membrane constituents, collagens and ECM structural constituents, ECM proteases, ECM protease inhibitors, other ECM molecules). Some low/non-expressed genes were excluded from analysis, and the relative expression of others is shown (Figure 9). While many genes associated with adhesion and matrix production were analyzed, significant expression differences are only shown for some (Figure 9, Table 1). SMG led to similar differences in osteocommitted and intact cells. In the same cases, the differences were more pronounced in osteocommitted MSCs (Table 1). Thus, in this group, significant changes of more than 1.5-fold were confirmed for 9 genes (*COL11A1*, *CTNNB1*, *HAS1*, *ITGA3*, *ITGB1*, *LAMA3*, *MMP1*, *MMP11*, and *TNC*). In intact cells, changes were shown only for 4 genes (*COL11A1*, *CTNND1*, *TIMP3*, and *TNC*). However, the directions of the differences were similar.

## 3. Discussion

The 10-day RPM exposure of MSCs (intact and osteocommitted) did not cause significant changes in morphology, immunophenotype, and cell viability. It should be mentioned that CD90, CD73, and CD105 expression decreased after osteocommittment. At the same time, the 10 days of SMG did not shift these expression patterns. It is known that integrins α2 and aV/β3 are involved in osteogenic differentiation, mediating signaling of MAPK and PI3K kinases that are required for the activation of master osteogenesis regulator runt-related transcription factor 2 (RUNX2) [43,44]. The decreased expression of these molecules may be compensatory negative feedback to osteogenic stimulation. A similar effect has been previously demonstrated [45]. The important role of CD90 and CD73 in osteo-differentiation was demonstrated in vivo since relevant knockout mice exhibit signs of osteopenia phenotype [46,47].

We observed the reduced level of collagenous proteins in MCS ECM after 10 days of SMG. Preliminary osteocommitment partly prevented the reduction in collagen content under SMG but did not eliminate the attenuation of collagens in the ECM in comparison with static control. Decrease of collagen production was demonstrated during exposure cultured MSCs to SMG and osteogenic stimuli [48]. These fibers constitute up to 90% of bone organics and determine the strength of tissues and mechanical resistance to stretching. ECM collagen fibers stimulate MSC osteogenesis due to mitogen-activated protein kinase (MAPK) signaling via integrins containing the β1 subunit [49]. Reduced collagen production by MSCs may cause the decrease in osteogenesis and contribute to the development of osteopenia during spaceflights.

The experimental results concerning gene expression in osteogenic precursors supported the possibility of decreased collagen production under microgravity. For example, 2–7-day exposure aboard SJ-10 satellite caused a downregulation in several collagen genes in bone marrow MSCs stimulated to undergo osteogenic differentiation [50]. A similar effect was revealed after 7 days of RPM exposure of osteoblasts [51]. COL1A1 expression as well as that of a number of other markers of osteogenic differentiation were downregulated in two types of cell lines during cultivation on board of ISS for 14 days [52]. It should be mentioned that under gravitational unloading, along with other signs of the bone imbalance, inhibition of collagen production was shown [53,54].

Simultaneously with a decrease in collagen level, we observed an increase of proteoglycans in the ECM after 10 days of SMG. These changes in ECM components can enhance its regulatory role because the deposition of VEGF, IGF, FGF, and other growth factors [55,56]. It can be assumed that a similar effect in vivo will result in the decomposition of proteoglycans accumulated during spaceflight and the release of growth factors after returning to normal gravity. It has been shown that proteoglycan overage in bone tissue occurs in some types of osteogenesis imperfecta and makes mineralization difficult [4].

Analysis of MSC transcriptomic profiles revealed moderate changes in different groups of ECM-associated genes. Among the 16 ITG genes, only ITGA3 and ITGB1 were altered after 10 days of SMG. It should be mentioned that short-term SMG caused impaired cell adhesion in vitro and transcriptomic shifts of adhesion molecules [42]. It is known that each of the integrin subunits is encoded by an individual gene and, therefore, decreased expression of one integrin subunit can attenuate the cell–matrix interaction. Subunits α5 and β1 associate to form fibronectin receptors, while α3 and β1 form laminin receptors [57]. Downregulation of α3 and α5 subunits may result in reduced numbers of laminin and fibronectin receptors and, therefore, in a decrease in the adhesion of osteocommitted MSCs. The attenuation of adhesion in intact MSCs may be due to an upregulation of TNC, since an excess of tenascin has been shown to attenuate cell adhesion to fibronectin. In addition, the interaction of tenascin with fibronectin stimulated the expression of a number of protease genes [58,59].

Moreover, the α3 subunit is responsible for activating the PI3K-Akt signaling pathway and is associated with focal adhesion kinase (FAK) [60,61]. At the same time, the β1 subunit is associated with MAPK signaling [40]. These signaling pathways are involved in osteogenic differentiation. It is possible that the integrin-mediated effect on these pathways could be compensated. A similar result was obtained after evaluation of the expression of anchor proteins (catenins) associated with adhesion. CTNNB1 expression was upregulated in osteocommitted MSCs, while CTNND1 was upregulated in intact cells after 10 days of SMG.

ECM remodeling comprises several simultaneous processes, including synthesis and degradation of different macromolecules. In our work, it was shown that intact and osteocommitted MSCs altered protease production in a similar way to under simulated microgravity. In both cases, an increase in cathepsin A, cathepsin D, and MMP3 secretion was detected. In addition, the level of MMP2 secretion increased in intact MSCs, while it decreased in osteo-MSC. This could be the reason for a lower decrease in collagenous proteins in osteo-MSCs. The activation of protease secretion under microgravity can lead to increased proteolysis and be one of bone resorption factors in vivo. MMP1 breaks down collagen types I, II, and III. MMP2 is specifically active against collagen IV, a major component of basal membranes. MMP3 is involved in the degradation of collagen types II, III, IV, IX, and X, proteoglycans, and fibronectin. This enzyme can also act as an activator of MMP1, MMP7, and MMP9, which makes it an important participant in connective tissue remodeling [62,63]. A similar upregulation of MMP1 and MMP3 in fibroblasts during 3-day SMG was shown by Buken et al. [37].

Cathepsins are lysosomal proteases, most of which have maximal activity at low pH. The degradation of intracellular proteins, hormones, and growth factors, and the regulation of apoptosis are the main physiological functions of cathepsin D. In addition, cathepsins are able to activate proteases. Cathepsin A is involved in hydrolysis of sialic acid in glycoproteins, thus playing a role in their degradation [64]. This may be especially important for the production of bone sialoprotein, involved in bone ECM mineralization. The study after 15-day spaceflight also demonstrated an upregulation of MMP1, MMP3, and MMP10 in the murine bone marrow MSCs, which indicated an activation of bone resorption and suggested the involvement of progenitor cells [10].

The family of metallopeptidase inhibitors (TIMP) is one of the important components for regulation of ECM degradation. In intact MSCs, the expression of TIMP1 and TIMP3 were downregulated after 10 days of SMG, which suggests the probable increased activity of MMPs. It is known that TIMP3 inhibits a wide range of matrix metalloproteinases such as MMP1, MMP2, MMP3, MMP7, MMP9, MMP13, MMP14, and MMP15 [65]. The effect of protease inhibitor reduction can be assumed to prevail due to the fact that each of them is capable of inhibiting more than one MMP. This is also indicated by a decrease in MSC collagenous proteins under SMG, since MMPs do respond for the proteolysis of collagens. It should be mentioned that in osteo-MSCs, the TIMP3 expression decreased is less pronounced under SMG. This may explain ECM collagen maintenance in these cells versus intact MSCs.

RPM exposure for 10 days induces MSC transcriptomic changes, which can lead to a weakening of adhesion to the matrix and slowdown in osteogenic differentiation. An upregulation of TNC expression after RPM exposure should be noted. Similar effects were observed in other experimental studies [66,67]. This is of particular interest since an excess of tenascin weakens cell adhesion to fibronectin [58]. Gene expression changes which can negatively affect cell adhesion under 96 h RPM exposure were shown [34]. After 10-day exposure, we did not find significant changes in the expression of molecules responsible for cell adhesion. Upregulation of TNC in MSCs under microgravity could activate matrix protease production, since it was shown that the interaction of tenascin with fibronectin stimulates the gene expression of proteases [59]. Apparently, there is a relationship between tenascin, protease expression, and cell adhesion under microgravity, and this issue requires further study.

Thus, after 10 days of SMG, the activation of ECM proteolytic processes was more pronounced in intact MSCs in comparison with osteocommited progeny. This is possible due to the downregulation of protease inhibitor encoding genes and increased secretion of MMPs. In addition, a 10-day RPM exposure provoked transcriptomic changes that can subsequently result in attenuated cell adhesion to the matrix. In the case of osteocommitted MSCs, this may happen due to a downregulation in integrin subunit genes, while in intact MSCs, it may occur due to an upregulation of tenascin. The attenuated adhesion to collagens may also be the reason for the inhibition of osteogenic differentiation via MAPK signaling. Thus, mesenchymal stromal cells of different commitment would differently contribute to the ECM degradation process of bone tissue during spaceflight. Further in vitro and in vivo studies are needed to support and extend the above findings.

## 4. Materials and Methods

Adipose tissue samples were obtained from the multidisciplinary clinic (Moscow, Russia) in the frame of scientific agreement. Samples were processed using the guidelines specifically approved by the Biomedicine Ethics Committee of the Institute of Biomedical Problems, Russian Academy of Sciences, Permit #314/MCK/09/03/13). Mesenchymal stromal cells (MSCs) were isolated using a standard method described by [68]. The isolated cells were stained with antibody against stromal markers CD90, CD73, CD105, and CD44 (BD Biosciences, San Jose, CA, USA) and were analyzed using an Accuri C6 flow cytometer (BD Biosciences, San Jose, CA, USA). The cells were expanded in α-MEM (Gibco, Life Technologies, Carlsbad, CA, USA) with 50 U/mL penicillin–streptomycin (PanEco, Moscow, Russia), and 10% fetal bovine serum (FBS) (HyClone, Logan, UT, USA) at standard conditions (5% CO_2_, 37 °C). Subculture was done at 80–90% confluence of the cell layer.

To induce adipogenic differentiation, the medium was supplemented with 0.5 mM isobutyl methylxanthine, 1 μM dexamethasone, 10 μg/mL insulin, and 200 μM indomethacin (Sigma, St. Louis, MO, USA). Adipogenic differentiation was assessed by the evaluation of cytoplasmic oil-red-O-stained lipid droplets (Millipore, Bedford, MA, USA).

To induce osteogenic differentiation, complete α-MEM was supplemented with 10 nM dexamethasone, 10 mM glycerol-2-phosphate, and 0.2 mM L-ascorbic acid 2-phosphate (Sigma, St. Louis, MO, USA). Osteogenic differentiation was confirmed using an alkaline phosphatase kit (Sigma-Aldrich, St. Louis, MO, USA) and alizarin red staining of the mineralized matrix components (Millipore, Bedford, MA, USA). The dye was extracted by DMSO and absorbance was measured using a plate photometer PR 2100 (Bio-Rad, USA) at 405 nm wavelength.

A desktop random positioning machine (RPM) (Dutch Space, Leiden, The Netherlands) was used to simulate the effects of microgravity. The speed (53–65 deg/s) and direction of the device rotation were randomized by dedicated control software at the computer user interface. The maximum distance between the cell monolayer and the center of rotation was 7.5 cm. The gravity value averaged 10^−3^–10^−2^ g [69].

The cells were plated in culture flask (surface area: 25 cm^2^, volume: 50 mL, Cellstar, Greiner Bio-One, Frickenhausen, Germany) at a density of 3000 cells. The osteogenic inducers were added in 2 of 4 culture flasks after MSCs reached 80–90% confluence. The inducers were removed from the medium after 7 days of cultivation. Flasks were filled with α-MEM (Gibco, Life Technologies, Carlsbad, CA, USA) with 50 U/mL penicillin–streptomycin (PanEco, Moscow, Russia), and 10% FBS (HyClone, Logan, UT, USA) without air bubbles to prevent sloshing of the medium and shear stress. The flasks were fixed on the RPM platform. The RPM was placed in a thermostat at 37 °C for 10 days.

To study MSC viability, cells were stained with annexin and propidium iodide using the Annexin V–FITC kit (Immunotech, France) according to the manufacturer’s instructions. Cells were analyzed using an Accuri C6 flow cytometer (BD Biosciences, San Jose, CA, USA). For cell size and structure analysis, flow cytometric forward scatter (FSC) and side scatter (SSC) density plots were applied.

The cells were stained with antibody against surface markers CD90, CD73, CD105, CD29, CD44, CD49a, CD49b, CD49e, CD51/61, and CD54 (BD Biosciences, San Jose, CA, USA) and were analyzed using an Accuri C6 flow cytometer (BD Biosciences, San Jose, CA, USA).

To assess the content of ECM components after RPM exposure, we used dyes that selectively bind to collagenous protein and non-collagenous proteins—Sirius Red F3BA and Fast Green FCF (Sigma, USA), respectively. We dissolved dyes in 1.2% picric acid at a ratio of 1:1000. The solution was poured into flasks with cells and incubated on a shaker for 30 min with a rotation of 60 rpm. An Eclipse TiU light microscope (Nikon, Tokyo, Japan) was used for visual assay of the staining of cultures with histological dyes. After that, the bound dyes were extracted with a mixture of methanol with 25 mM NaOH (1:1) and optical density was compared on a PR 2100 plate photometer (Bio-Rad, USA) at 550 nm for Sirius Red and 605 nm for Fast Green.

The conditioned medium (CM) was collected after experiments, centrifuged at 2500× *g* to remove cell debris, and stored at −80 °C (low temperature freezer, Sanyo, Osaka, Japan). To detect 35 human proteases (ADAM8, ADAM9, ADAMTS1, ADAMTS13, cathepsin A, cathepsin B, cathepsin C, cathepsin D, cathepsin E, cathepsin L, cathepsin S, cathepsin V, cathepsin X/Z/P, DPPIV/CD26, kallikrein 3/PSA, kallikrein 5, kallikrein 6, kallikrein 7, kallikrein 10, kallikrein 11, kallikrein 13, MMP1, MMP2, MMP3, MMP7, MMP8, MMP9, MMP10, MMP12, MMP13, neprilysin/CD10, presenilin, proprotein convertase 9, proteinase 3, uPA/urokinase) CM was analyzed using Proteome Profiler Human Protease Array Kit (R&D Systems, Inc., Minneapolis, MN, USA) according to the manufacturer’s instructions. The data were analyzed using Image Lab Software Version 5.0 (Bio-Rad, Hercules, CA, USA).

To evaluate gene expression, total RNA was extracted with QIAzol reagent (Qiagen, Hilden, Germany) and purified using the phenol/chloroform technique. The quality and concentration of RNA samples were estimated using a Nanodrop ND-2000c (Thermo Scientific, Waltham, MA, USA). Ambion DNase I (RNase-free) (Thermo Fisher Scientific, Waltham, MA, USA) was used for genomic DNA degradation. Reverse transcription was performed using the MMLV RT Kit (Eurogene, Moscow, Russia) according to the manufacturer’s protocol. Expression of 84 matrix/adhesion associated genes was analyzed using RT^2^ Profiler PCR Array—Human Extracellular Matrix & Adhesion Molecules (Qiagen). This gene profiler included cell adhesion molecules (transmembrane receptors—*CD44*, *CDH1 (E-Cadherin)*, *HAS1*, *ICAM1*, *ITGA1*, *ITGA2*, *ITGA3*, *ITGA4 (CD49D)*, *ITGA5*, *ITGA6*, *ITGA7*, *ITGA8*, *ITGAL*, *ITGAM*, *ITGAV*, *ITGB1*, *ITGB2*, *ITGB3*, *ITGB4*, *ITGB5*, *MMP14*, *MMP15*, *MMP16*, *NCAM1*, *PECAM1*, *SELE*, *SELL (LECAM-1)*, *SELP*, *SGCE*, *SPG7*, *VCAM1*; cell–cell adhesion—*CD44*, *CDH1 (E-Cadherin)*, *COL11A1*, *COL14A1*, *COL6A2*, *CTNND1*, *ICAM1*, *ITGA8*, *VCAM1*; cell–ECM adhesion—*ADAMTS13*, *CD44*, *ITGA1*, *ITGA2*, *ITGA3*, *ITGA4 (CD49D)*, *ITGA5*, *ITGA6*, *ITGA7*, *ITGA8*, *ITGAL*, *ITGAM*, *ITGAV*, *ITGB1*, *ITGB2*, *ITGB3*, *ITGB4*, *ITGB5*, *SGCE*, *SPP1*, *THBS3*; other cell adhesion molecules—*ANOS1*, *CCN2*, *CLEC3B*, *CNTN1*, *COL12A1*, *COL15A1*, *COL16A1*, *COL5A1*, *COL6A1*, *COL7A1*, *COL8A1*, *CTNNA1*, *CTNNB1*, *CTNND2*, *FN1*, *LAMA1*, *LAMA2*, *LAMA3*, *LAMB1*, *LAMB3*, *LAMC1*, *THBS1 (TSP-1)*, *THBS2*, *TNC*, *VCAN*, *VTN*) and ECM molecules (basement membrane constituents—*COL4A2*, *COL7A1*, *LAMA1*, *LAMA2*, *LAMA3*, *LAMB1*, *LAMB3*, *LAMC1*, *SPARC*; collagens & ECM structural constituents—*ANOS1*, *COL11A1*, *COL12A1*, *COL14A1*, *COL15A1*, *COL16A1*, *COL1A1*, *COL4A2*, *COL5A1*, *COL6A1*, *COL6A2*, *COL7A1*, *COL8A1*, *FN1*; ECM proteases—*ADAMTS1*, *ADAMTS13*, *ADAMTS8*, *MMP1*, *MMP10*, *MMP11*, *MMP12*, *MMP13*, *MMP14*, *MMP15*, *MMP16*, *MMP2*, *MMP3*, *MMP7*, *MMP8*, *MMP9*, *SPG7*, *TIMP1*; ECM protease inhibitors—*ANOS1*, *COL7A1*, *THBS1 (TSP-1)*, *TIMP1*, *TIMP2*, *TIMP3*; other ECM molecules—*CCN2*, *CLEC3B*, *ECM1*, *HAS1*, *SPP1*, *TGFBI*, *THBS2*, *THBS3*, *TNC*, *VCAN*, *VTN*). The resulting cDNA was mixed with RT2 SYBR Green/ROX PCR Master Mix (Qiagen) and added to 96-well plates according to the manufacturer’s protocol. The expression levels of five housekeeping genes (*ACTB*, *B2M*, *GAPDH*, *HPRT*, and *RPLP0*) were used for reference. Expression of the genes *RUNX2*, *ALPL*, *OPG*, *SP7*, and *BGLAP* was analyzed using Qiagen primers (Qiagen). The cDNA was mixed with qPCRmix-HS SYBR (Eurogene, Moscow, Russia) and added to 96-well plates. The expression levels of *RPLP0* and *HPRT* were used for reference. qPCR was performed using the Mx3000P system (Stratagene, San Diego, CA, USA). Relative gene expression was calculated using the 2^−ΔΔCt^ method [70].

A minimum of three independent experiments were carried out for each assay. Analysis of group differences was performed by nonparametric Mann–Whitney test for independent samples using STATISTICA 10 software (Statsoft, Tulsa, OK, USA). A level of *p* < 0.05 was accepted as statistically significant.

## Figures and Tables

**Table 1:** Relative expression of matrix/adhesion associated genes after 10 days of RPM exposure of intact (Int) and osteocommitted (Ost) MSCs.

Gene	Description	Fold Change (Mean ± SD)	
		RPM (Int) vs. Stat (Int)	RPM (Ost) vs. Stat (Ost)
*COL11A1*	Collagen, type XI, alpha 1	1.48 ± 0.39 *	1.92 ± 0.80 *
*CTNNB1*	Catenin, beta 1	1.05 ± 0.59	1.82 ± 1.10 *
*CTNND1*	Catenin, delta 1	1.56 ± 0.60 *	0.70 ± 0.26
*HAS1*	Hyaluronan synthase 1	0.90 ± 0.13	0.55 ± 0.37 *
*ITGA3*	Integrin, alpha 3 (antigen CD49C)	0.72 ± 0.35	0.53 ± 0.23 *
*ITGB1*	Integrin, beta 1	1.13 ± 0.26	1.56 ± 0.45 *
*LAMA3*	Laminin, alpha 3	0.94 ± 0.69	0.63 ± 0.25 *
*MMP1*	Matrix metallopeptidase 1	1.77 ± 1.53	0.63 ± 0.10 *
*MMP11*	Matrix metallopeptidase 11	0.82 ± 0.13	0.51 ± 0.05 *
*TIMP3*	TIMP metallopeptidase inhibitor 3	0.66 ± 0.13 *	0.75 ± 0.24
*TNC*	Tenascin C	2.23 ± 0.99 *	2.16 ± 1.55 *

* The significantly changed genes (more than 1.5-fold, *p* ≤ 0.05) are presented; *n* ≥ 3.

## Data Availability

Not applicable.

## References

[1] Kadler K.E., Baldock C., Bella J., Boot-Handford R.P. (2007). Collagens at a glance. J. Cell Sci..

[2] Bülow H.E., Hobert O. (2006). The Molecular Diversity of Glycosaminoglycans Shapes Animal Development. Annu. Rev. Cell Dev. Biol..

[3] Hyldig K., Riis S., Pennisi C.P., Zachar V., Fink T. (2017). Implications of Extracellular Matrix Production by Adipose Tissue-Derived Stem Cells for Development of Wound Healing Therapies. Int. J. Mol. Sci..

[4] Sarathchandra P., Cassella J.P., Ali S.Y. (2002). Ultrastructural localization of proteoglycans in bone in osteogenesis imperfecta as demonstrated by Cuprolinic Blue staining. J. Bone Miner. Metab..

[5] Argentati C., Morena F., Tortorella I., Bazzucchi M., Porcellati S., Emiliani C., Martino S. (2019). Insight into Mechanobiology: How stem cells feel mechanical forces and orchestrate biobical functions. Int. J. Mol. Sci..

[6] Mott J.D., Werb Z. (2004). Regulation of matrix biology by matrix metalloproteinases. Curr. Opin. Cell Biol..

[7] Oganov V.S., Bakulin A.V., Novikov V.E., Kabitskaia O.E., Murashko L.M. (2006). Characteristics and patterns of the human bone reactions to microgravity. Aerosp. Environ. Med..

[8] Grimm D., Grosse J., Wehland M., Mann V., Reseland J.E., Sundaresan A., Corydon T.J. (2016). The impact of microgravity on bone in humans. Bone.

[9] Stavnichuk M., Mikolajewicz N., Corlett T., Morris M., Komarova S.V. (2020). A systematic review and meta-analysis of bone loss in space travelers. NPJ Microgravity.

[10] Blaber E.A., Dvorochkin N., Lee C., Alwood J.S., Yousuf R., Pianetta P., Globus R.K., Burns B.P., Almeida E.A.C. (2013). Microgravity Induces Pelvic Bone Loss through Osteoclastic Activity, Osteocytic Osteolysis, and Osteoblastic Cell Cycle Inhibition by CDKN1a/p21. PLoS ONE.

[11] Camirand A., Goltzman D., Gupta A., Kaouass M., Panda D., Karaplis A. (2016). The Role of Parathyroid Hormone-Related Protein (PTHrP) in Osteoblast Response to Microgravity: Mechanistic Implications for Osteoporosis Development. PLoS ONE.

[12] Klein-Nulend J., Bacabac R., Veldhuijzen J., Van Loon J. (2003). Microgravity and bone cell mechanosensitivity. Adv. Space Res..

[13] Maier J.A.M., Cialdai F., Monici M., Morbidelli L. (2015). The Impact of Microgravity and Hypergravity on Endothelial Cells. BioMed Res. Int..

[14] Ludtka C., Silberman J., Moore E., Allen J.B. (2021). Macrophages in microgravity: The impact of space on immune cells. NPJ Microgravity.

[15] Wuest S.L., Richard S., Kopp S., Grimm D., Egli M. (2015). Simulated Microgravity: Critical Review on the Use of Random Positioning Machines for Mammalian Cell Culture. BioMed Res. Int..

[16] Chen Z., Luo Q., Yuan L., Song G. (2016). Microgravity directs stem cell differentiation. Histol. Histopathol..

[17] Grimm D., Wise P., Lebert M., Richter P., Baatout S. (2011). How and why does the proteome respond to microgravity?. Expert Rev. Proteom..

[18] Cristofaro F., Pani G., Pascucci B., Mariani A., Balsamo M., Donati A., Mascetti G., Rea G., Rizzo A.M., Visai L. (2019). The NATO project: Nanoparticle-based countermeasures for microgravity-induced osteoporosis. Sci. Rep..

[19] Carmeliet G., Vico L., Bouillon R. (2001). Space flight: A challenge for normal bone homeostasis. Crit. Rev. Eukaryot. Gene Expr..

[20] Gershovich P.M., Gershovich I.G., Buravkova L.B. (2014). The effects of simulated microgravity on the pattern of gene expression in human bone marrow mesenchymal stem cells under osteogenic differentiation. Fiziologiia Cheloveka.

[21] Buravkova L.B., Gershovich Y.G., Grigorev A.I. (2010). Sensitivity of stromal precursor cells of different commitment to simulated microgravity. Dokl. Biol. Sci..

[22] Katsaras G.N., Lambrou G.I. (2019). Gene Expression in Osteoblasts and Osteoclasts Under Microgravity Conditions: A Systematic Review. Curr. Genom..

[23] Buravkova L., Gershovich P., Gershovich J., Grigor’Ev A. (2010). Mechanisms of Gravitational Sensitivity of Osteogenic Precursor Cells. Acta Nat..

[24] Li L., Zhang C., Chen J.-L., Hong F.-F., Chen P., Wang J.-F. (2018). Effects of simulated microgravity on the expression profiles of RNA during osteogenic differentiation of human bone marrow mesenchymal stem cells. Cell Prolif..

[25] Muncie J.M., Weaver V.M. (2018). The Physical and Biochemical Properties of the Extracellular Matrix Regulate Cell Fate. Curr. Top. Dev. Biol..

[26] Ozdil B., Güler G., Acikgoz E., Kocaturk D.C., Aktug H. (2020). The effect of extracellular matrix on the differentiation of mouse embryonic stem cells. J. Cell. Biochem..

[27] Jha A.K., Jackson W.M., Healy K.E. (2014). Controlling Osteogenic Stem Cell Differentiation via Soft Bioinspired Hydrogels. PLoS ONE.

[28] Fernández-Pernas P., Barrachina L., Marquina M., Rodellar C., Arufe M., Costa C. (2020). Mesenchymal stromal cells for articular cartilage repair: Preclinical studies. Eur. Cells Mater..

[29] Barba M., Cicione C., Bernardini C., Michetti F., Lattanzi W. (2013). Adipose-Derived Mesenchymal Cells for Bone Regereneration: State of the Art. BioMed Res. Int..

[30] Murphy M.B., Moncivais K., Caplan A.I. (2013). Mesenchymal stem cells: Environmentally responsive therapeutics for regenerative medicine. Exp. Mol. Med..

[31] Ulbrich C., Wehland M., Pietsch J., Aleshcheva G., Wise P., Van Loon J., Magnusson N.E., Infanger M., Grosse J., Eilles C. (2014). The Impact of Simulated and Real Microgravity on Bone Cells and Mesenchymal Stem Cells. BioMed Res. Int..

[32] Ratushnyy A., Yakubets D., Andreeva E., Buravkova L. (2019). Simulated microgravity modulates the mesenchymal stromal cell response to inflammatory stimulation. Sci. Rep..

[33] Buravkova L.B., Gershovich P.M., Gershovich J.G., Grigoriev A.I. (2013). Microgravity and Mesenchymal Stem Cell Response. Curr. Biotechnol..

[34] Ratushnyy A.Y., Buravkova L.B. (2017). Expression of focal adhesion genes in mesenchymal stem cells under simulated microgravity. Dokl. Biochem. Biophys..

[35] Aleshcheva G., Sahana J., Ma X., Hauslage J., Hemmersbach R., Egli M., Infanger M., Bauer J., Grimm D. (2013). Changes in Morphology, Gene Expression and Protein Content in Chondrocytes Cultured on a Random Positioning Machine. PLoS ONE.

[36] Ebnerasuly F., Hajebrahimi Z., Tabaie S.M., Darbouy M. (2018). Simulated Microgravity Condition Alters the Gene Expression of some ECM and Adhesion Molecules in Adipose Derived Stem Cells. Int. J. Mol. Cell. Med..

[37] Buken C., Sahana J., Corydon T.J., Melnik D., Bauer J., Wehland M., Krüger M., Balk S., Abuagela N., Infanger M. (2019). Morphological and Molecular Changes in Juvenile Normal Human Fibroblasts Exposed to Simulated Microgravity. Sci. Rep..

[38] Makihira S., Kawahara Y., Yuge L., Mine Y., Nikawa H. (2008). Impact of the microgravity environment in a 3-dimensional clinostat on osteoblast- and osteoclast-like cells. Cell Biol. Int..

[39] Pardo S.J., Patel M.J., Sykes M.C., Platt M.O., Boyd N.L., Sorescu G.P., Xu M., Van Loon J.J.W.A., Wang M.D., Jo H. (2005). Simulated microgravity using the Random Positioning Machine inhibits differentiation and alters gene expression profiles of 2T3 preosteoblasts. Am. J. Physiol. Physiol..

[40] Dominici M., Le Blanc K., Mueller I., Slaper-Cortenbach I., Marini F.C., Krause D.S., Deans R.J., Keating A., Prockop D.J., Horwitz E.M. (2006). Minimal criteria for defining multipotent mesenchymal stromal cells. The International Society for Cellular Therapy position statement. Cytotherapy.

[41] Byers B.A., Pavlath G.K., Murphy T.J., Karsenty G., Garcia A.J. (2002). Cell-Type-Dependent Up-Regulation of In Vitro Mineralization After Overexpression of the Osteoblast-Specific Transcription Factor Runx2/Cbfa1. J. Bone Miner. Res..

[42] Ratushny A., Yakubets D., Zhivodernikov I., Buravkova L. (2017). Adhesion molecules of multipotent mesenchymal stromal cells obtained from adipose tissue during simulation of the effects of microgravity. Aerosp. Environ. Med..

[43] Kundu A.K., Khatiwala C.B., Putnam A.J. (2009). Extracellular Matrix Remodeling, Integrin Expression, and Downstream Signaling Pathways Influence the Osteogenic Differentiation of Mesenchymal Stem Cells on Poly(Lactide-Co-Glycolide) Substrates. Tissue Eng. Part A.

[44] Hu H.-M., Yang L., Wang Z., Liu Y.-W., Fan J.-Z., Fan J., Liu J., Luo Z.-J. (2013). Overexpression of integrin a2 promotes osteogenic differentiation of hBMSCs from senile osteoporosis through the ERK pathway. Int. J. Clin. Exp. Pathol..

[45] Lee H.M., Seo S., Kim J., Kim M.K., Seo H., Kim K.S., Jang Y., Ryu C.J. (2020). Expression dynamics of integrin α2, α3, and αV upon osteogenic differentiation of human mesenchymal stem cells. Stem Cell Res. Ther..

[46] Picke A.-K., Campbell G.M., Blüher M., Krügel U., Schmidt F.N., Tsourdi E., Winzer M., Rauner M., Vukicevic V., Busse B. (2018). Thy-1 (CD90) promotes bone formation and protects against obesity. Sci. Transl. Med..

[47] Bradaschia-Correa V., Josephson A.M., Egol A.J., Mizrahi M.M., Leclerc K., Huo J., Cronstein B.N., Leucht P. (2017). Ecto-5′-nucleotidase (CD73) regulates bone formation and remodeling during intramembranous bone repair in aging mice. Tissue Cell.

[48] Uddin S.M.Z., Qin Y.-X. (2013). Enhancement of Osteogenic Differentiation and Proliferation in Human Mesenchymal Stem Cells by a Modified Low Intensity Ultrasound Stimulation under Simulated Microgravity. PLoS ONE.

[49] Franceschi R.T., Xiao G. (2003). Regulation of the osteoblast-specific transcription factor, Runx2: Responsiveness to multiple signal transduction pathways. J. Cell. Biochem..

[50] Zhang C., Li L., Jiang Y., Wang C., Geng B., Wang Y., Chen J., Liu F., Qiu P., Zhai G. (2018). Space microgravity drives transdifferentiation of human bone marrow-derived mesenchymal stem cells from osteogenesis to adipogenesis. FASEB J..

[51] Meyers V.E., Zayzafoon M., Gonda S.R., Gathings W.E., McDonald J.M. (2004). Modeled microgravity disrupts collagen I/integrin signaling during osteoblastic differentiation of human mesenchymal stem cells. J. Cell. Biochem..

[52] Colucci S., Colaianni G., Brunetti G., Ferranti F., Mascetti G., Mori G., Grano M. (2020). Irisin prevents microgravity-induced impairment of osteoblast differentiation in vitro during the space flight CRS-14 mission. FASEB J..

[53] Caillot-Augusseau A., Vico L., Heer M., Voroviev D., Souberbielle J.C., Zitterman A., Alexandre C., Lafage-Proust M.H. (2000). Space flight is associated with rapid decreases of undercarboxylated osteocalcin and increases of markers of bone resorption without changes in their circadian variation: Observations in two cosmonauts. Clin. Chem..

[54] Vico L., Hinsenkamp M., Jones D., Marie P.J., Zallone A., Cancedda R. (2001). Osteobiology, strain, and microgravity. Part II: Studies at the tissue level. Calcif. Tissue Int..

[55] Zandi N., Mostafavi E., Shokrgozar M.A., Tamjid E., Webster T.J., Annabi N., Simchi A. (2019). Biomimetic proteoglycan nanoparticles for growth factor immobilization and delivery. Biomater. Sci..

[56] Chun S.Y., Lim J.O., Lee E.H., Han M.-H., Ha Y.-S., Lee J.N., Kim B.S., Park M.J., Yeo M., Jung B. (2019). Preparation and Characterization of Human Adipose Tissue-Derived Extracellular Matrix, Growth Factors, and Stem Cells: A Concise Review. Tissue Eng. Regen. Med..

[57] Luo B.-H., Springer T.A. (2006). Integrin structures and conformational signaling. Curr. Opin. Cell Biol..

[58] Tremble P., Chiquet-Ehrismann R., Werb Z. (1994). The extracellular matrix ligands fibronectin and tenascin collaborate in regulating collagenase gene expression in fibroblasts. Mol. Biol. Cell.

[59] Probstmeier R., Pesheva P. (1999). Tenascin-C inhibits 1 integrin-dependent cell adhesion and neurite outgrowth on fibronectin by a disialoganglioside-mediated signaling mechanism. Glycobiology.

[60] Hu J.K., Du W., Shelton S.J., Oldham M.C., DiPersio C.M., Klein O.D. (2017). A FAK-YAP-mTOR signaling axis regulates stem cell-based tissue renewal in mice. Cell Stem Cell.

[61] Jiao Y., Li Y., Liu S., Chen Q., Liu Y. (2019). ITGA3 serves as a diagnostic and prognostic biomarker for pancreatic cancer. OncoTargets Ther..

[62] Docherty A.J., Murphy G. (1990). The tissue metalloproteinase family and the inhibitor TIMP: A study using cDNAs and recombinant proteins. Ann. Rheum. Dis..

[63] Sun S., Bay-Jensen A.-C., Karsdal M.A., Siebuhr A.S., Zheng Q., Maksymowych W.P., Christiansen T.G., Henriksen K. (2014). The active form of MMP-3 is a marker of synovial inflammation and cartilage turnover in inflammatory joint diseases. BMC Musculoskelet. Disord..

[64] Patel S., Homaei A., El-Seedi H.R., Akhtar N. (2018). Cathepsins: Proteases that are vital for survival but can also be fatal. Biomed. Pharmacother..

[65] Fan D., Kassiri Z. (2020). Biology of Tissue Inhibitor of Metalloproteinase 3 (TIMP3), and Its Therapeutic Implications in Cardiovascular Pathology. Front. Physiol..

[66] He L., Pan S., Li Y., Zhang L., Zhang W., Yi H., Song C., Niu Y. (2015). Increased proliferation and adhesion properties of human dental pulp stem cells in PLGA scaffolds via simulated microgravity. Int. Endod. J..

[67] Kraus A., Luetzenberg R., Abuagela N., Hollenberg S., Infanger M. (2019). Spheroid formation and modulation of tenocyte-specific gene expression under simulated microgravity. Muscle Ligaments Tendons J..

[68] Zuk P.A., Zhu M., Ashjian P., De Ugarte D.A., Huang J.I., Mizuno H., Alfonso Z.C., Fraser J.K., Benhaim P., Hedrick M.H. (2002). Human Adipose Tissue Is a Source of Multipotent Stem Cells. Mol. Biol. Cell.

[69] Van Loon J.J. (2007). Some history and use of the random positioning machine, RPM, in gravity related research. Adv. Space Res..

[70] Livak K.J., Schmittgen T.D. (2001). Analysis of relative gene expression data using real-time quantitative PCR and the 2(-Delta Delta C(T)). Methods.

